# Mitochondria as extracellular vesicle cargo in aging

**DOI:** 10.18632/aging.203358

**Published:** 2021-07-20

**Authors:** Nicole Noren Hooten, Michele K. Evans

**Affiliations:** 1 Laboratory of Epidemiology and Population Science, National Institute on Aging, National Institutes of Health, Baltimore, MD 21224, USA

**Keywords:** exosomes, extracellular vesicles, mitochondria, mitochondrial DNA, biomarker, aging, microvesicles, circulating cell-free mitochondrial DNA

**Figure 1:**
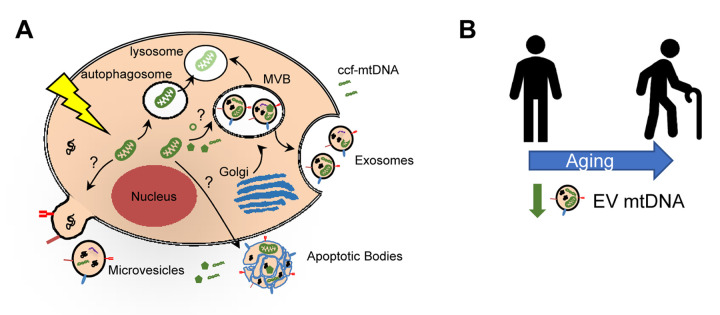
**Mitochondrial DNA in extracellular vesicles and association with human aging.** Damage to mitochondria can lead to release of mitochondrial components, including mitochondrial DNA (mtDNA). Circulating cell-free mtDNA (ccf-mtDNA) can be released into the circulation. It can also be encapsulated into extracellular vesicles (EVs). Three main types of EVs are indicated and the possible mechanism for incorporation of mitochondrial components. Alternatively, damaged mitochondria can be degraded via the mitophagy pathway. (B) Schematic representation showing decreased circulating EV mtDNA levels with human age.

Aging is associated with a state of chronic low-grade inflammation, termed ‘inflamm-ageing’, that likely contributes to many age-related pathologies including cardiovascular disease, chronic kidney disease, type 2 diabetes mellitus, cancer and dementia [1]. In fact, inflammation-related diseases account for more than 50% of worldwide deaths, stressing the importance of inflammation in driving age-related disease and mortality [1,2]. Many factors contribute to chronic inflammation in the elderly [1]. Cellular damage or stress can initiate a release of mitochondrial damage-associated molecular patterns (DAMPs). As part of this process, mitochondrial DNA (mtDNA) can be released into the extracellular space as circulating cell-free mitochondria DNA (ccf-mtDNA) (Figure 1A). Due to the similarities between mtDNA and bacterial DNA, this release can in turn elicit a sterile inflammatory response through activation of the innate immune system.

Recent attention has focused on detection and characterization of ccf-mtDNA in the blood. In general, higher plasma/serum levels of ccf-mtDNA have been reported in inflammatory-related diseases, and in response to acute tissue injury such as trauma, acute myocardial infarction, or sepsis [3]. The relationship between ccf-mtDNA and aging is more complex as one report showed an initial decline in ccf-mtDNA into middle-age and then a gradual increase after the fifth decade of life [4]. Individuals greater than 90 years of age with high levels of ccf-mtDNA had higher levels of the proinflammatory cytokines TNF-α, IL-6, IL-1ra and RANTES compared to individuals with low levels of ccf-mtDNA who also had lower levels of these cytokines [4]. Together these studies indicate that ccf-mtDNA may contribute to systemic chronic inflammation. However, we are only beginning to understand the molecular details of how ccf-mtDNA exists in the circulation. Thus far, most studies examine ccf-mtDNA in complex body fluids such as plasma and serum and little is known about the components to which they bind and whether these components may protect ccf-mtDNA from destruction in the circulation.

Given these gaps in the field, we recently explored whether plasma mtDNA can be encapsulated in extracellular vesicles (EVs) [5]. EVs are lipid-bound nano-sized vesicles that are secreted outside of cells into the circulation (Figure 1A). They contain bioactive molecules including proteins, nucleic acids and lipids and serve to protect cargo from degradation [6]. EVs play a variety of roles depending on cellular context and stimuli and include disposing of cellular debris and communicating between neighboring and distant cells [6]. We found that mtDNA can be encapsulated in EVs isolated from plasma (Figure 1A). This data is consistent with other reports showing that cells grown *in vitro* contain mtDNA in EVs and that mtDNA is present in plasma EVs from breast cancer patients with hormonal-resistance to therapy (reviewed in [7]).

Little is known about whether mtDNA is present in plasma EVs under normal physiological conditions or whether mitochondrial components are important functional cargo in EVs. To address this need, we isolated plasma EVs and analyzed mtDNA levels with human age. Individuals in this aging cohort had donated plasma at two different time points approximately 5 years apart, which enabled us to examine both cross-sectional and longitudinal changes. In both our cross-sectional and longitudinal analyses, EV mtDNA levels decreased with advancing age [5] (Figure 1B).

Mitochondrial dysfunction contributes to the aging process. A few recent studies have examined whether mitochondrial components may be functional cargo in EVs [7] (Figure 1A). These studies point to a potential mechanism whereby mtDNA in EVs can be transferred to recipient cells and elicit functional changes [7]. However, it is not fully understood whether this is a general mechanism or specific to certain cell types or stimuli. Nevertheless, these initial studies highlight the potential importance of mtDNA in EVs. To further address this, we examined whether EVs from young and old individuals with different mtDNA levels affect mitochondrial function. Cells treated with EVs from old individuals, which contain lower mtDNA levels, had significantly lower basal and maximal respiration than cells treated with EVs from young individuals [5]. These data suggest that EVs from old individuals may impair mitochondrial function.

Taken together, recent data indicate that mitochondrial components, including mtDNA, may be important EV cargo. This data also expands upon further, broadly characterizing EVs in diverse human populations in the context of aging. Given that age-dependent changes in EVs occur [5,8], it is important for age to be considered when utilizing EVs as diagnostic or prognostic markers of disease.
